# Prenatal Diagnosis and Evaluation of Sonographic Predictors for Intervention and Adverse Outcome in Congenital Pulmonary Airway Malformation

**DOI:** 10.1371/journal.pone.0150474

**Published:** 2016-03-15

**Authors:** Astrid Hellmund, Christoph Berg, Annegret Geipel, Meike Bludau, Andreas Heydweiller, Haitham Bachour, Andreas Müller, Annette Müller, Ulrich Gembruch

**Affiliations:** 1 Department of Obstetrics and Prenatal Medicine, University of Bonn, Bonn, Germany; 2 Division of Prenatal Medicine and Gynecologic Sonography, Department of Obstetrics and Gynecology, University of Cologne, Cologne, Germany; 3 Division of Pediatric Surgery, University of Bonn, Bonn, Germany; 4 Department of Neonatology, University of Bonn, Bonn, Germany; 5 Department of Pathology, University of Bonn, Bonn, Germany; Hospital de Especialidades del Niño y la Mujer de Queretaro, MEXICO

## Abstract

**Objective:**

To describe antenatal findings and evaluate prenatal risk parameters for adverse outcome or need for intervention in fetuses with congenital pulmonary airway malformation (CPAM).

**Methods:**

In our retrospective study all fetuses with a prenatal diagnosis of CPAM detected in our tertiary referral center between 2002 and 2013 were analyzed. Sonographic findings were noted and measurements of mass-to-thorax-ratio (MTR), congenital pulmonary airway malformation volume-ratio (CVR) and observed to expected lung-to head-ratio (o/e LHR) were conducted and correlated to fetal or neonatal morbidity and mortality and/or need for prenatal intervention.

**Results:**

67 fetuses with CPAM were included in the study. Hydropic fetuses were observed in 16.4% (11/67) of cases, prenatal intervention was undertaken in 9 cases; 7 pregnancies were terminated. The survival rate of non-hydropic fetuses with conservatively managed CPAM was 98.0% (50/51), the survival rate for hydropic fetuses with intention to treat was 42.9% (3/7). 10 (18.2%) children needed respiratory assistance. Fetuses with a CVR of <0.91 were significantly less likely to experience adverse outcome or need for prenatal intervention with sensitivity, specificity and positive/negative predictive value of 0.89, 0.71, 0.62 and 0.93, respectively. A MTR (mass-to-thorax-ratio) of < 0.51 had a positive predictive value of 0.54 and a negative predictive value of 0.96 of adverse events with a sensitivity of 0.95 and a specificity of 0.63. The negative predictive value for o/e LHR of 45% was 0.84 with sensitivity, specificity and positive predictive value of 0.73, 0.68 and 0.52, respectively.

**Conclusions:**

The majority of cases with CPAM have a favorable outcome. MTR and CVR are able to identify fetuses at risk, the o/e LHR is less sensitive.

## Introduction

Prenatal diagnosis of echogenic lung lesions can be achieved using high resolution ultrasound during the second trimester. The most common causative lesions are congenital pulmonary airway malformation (CPAM) and pulmonary sequestration (BPS). Few studies have described prognosis, prenatal and postnatal management of these lesions [1,2,3,4,5,6,7,8,9,10]. Though the majority of fetuses with CPAM usually have a favorable outcome, the prognosis of rare cases with large lesions complicated by hydrops and need for prenatal intervention is remarkably worse. In order to provide more information on the prognosis of these fetuses, we conducted a retrospective single-center study of 67 consecutive cases of CPAM over the last 12 years, focusing on intrauterine prognostic markers such as MTR, CVR and o/e LHR predicting need for prenatal intervention, respiratory assistance and fetal mortality.

## Methods

This is a retrospective study of data routinely achieved and anonymously analyzed. All participants (mothers of the fetuses) signed in a form, that the data may be used anonymously for research use. Our local ethics committee (Ethik Kommission der Medizinischen Fakultät der Universität Bonn) does not generate a protocol in this form of study but exempted us from approval in a formal waver. The mothers were contacted at the routinely performed sonography, not especially for the study. Sonographic findings were extracted from the medical records and stored in an anonymized data base by the authors, as well as information to postnatal therapy and outcome. After data extraction no identifying information was stored by the researchers. Our hospital gave permission to the publication of patient`s medical images.

The study was carried out between 2002 and 2013. In this period 59,082 cases were sent to our tertiary referral center for a detailed second trimester scan. The antenatal findings of 67 fetuses with prenatal diagnosis of CPAM stored in our computerized database were analyzed. Location and size of the lesion, presence of mediastinal shifting and associated malformations were noted. Follow up scans with regard to regression or progression of the lesion were included. All patients received a complete anatomic scan and Doppler sonography using ATL HDI 5000 and IU22, Philips (Hamburg, Germany) or Voluson E8, GE Healthcare (Solingen, Germany) devices. In cases of fetal intervention the different methods (single or repeated thoracocentesis, shunting) were noted as well as success of the intervention in the course of pregnancy. Shunting was performed with a double pigtail catheter (Harrison fetal bladder Stent Set, Cook Medical, Bloomington, IN, USA). The type of lesion was recorded using the classification described by Stocker [11]. To calculate the CVR (CPAM volume ratio), a volumetric index of mass size that allows for comparison of fetuses at different gestational ages [1], the length, width and depth of the mass were multiplied by a 0.52 correction factor and divided by the head circumference. Moreover the transverse diameter of the mass and the transverse diameter of the thorax were taken and the mass-to-thorax-ratio (MTR) was calculated [12]. Finally the observed to expected lung area to head circumference ratio, showing the LHR in relation to the fetal head circumference independant of the gestational age and usually performed as a prognostic marker in cases with congenital diaphragmatic hernia, was measured [13]. Furthermore adverse events during pregnancy, mode of delivery, postnatal respiratory adaptation of the newborns and long term outcome were analyzed based on obstetrical and pediatric charts. Postnatal confirmation of the diagnosis was obtained by chest x-ray, contrast computerized tomography or magnetic resonance imaging. In cases of postnatal surgery histopathological findings were analyzed.

Statistical analyses were performed with the Fisher exact test with the SPSS software package (SPSS Inc, Chicago IL). Receiver operating characteristic curves were calculated based on the CVR, MTR or o/e LHR in relationship to outcome and/or need for intervention. Adverse outcome/need for intervention was defined as perinatal death, termination of pregnancy, hydrops, need for prenatal intervention such as pleural drainage and/or thoracocentesis or need for postnatal respiratory assistance. Significance was defined as a probability value of < .05.

## Results

In the study period 67 fetuses (38 male/ 29 female fetuses) of CPAM were diagnosed, including three cases with hybrid lesions. Median gestational age at diagnosis was at 22 weeks (range 17–29). The microcystic, macrocystic and mixed type of lesion was diagnosed in 34 (50.7%), 14 (20.9%) and 19 (28.4%) cases, respectively. There was no predominance of left sided or right sided lesions (47.8% vs. 52.2%). Mediastinal shifting occured in 56 (83.6%) fetuses; the shift was discrete, moderate and severe in 21 (31.3%), 23 (34.3%) and 12 (17.9%) cases, respectively. Cases with macrocystic lesions showed predominately moderate (28.6%) and severe (57.1%) shifting.

**Fig 1:**
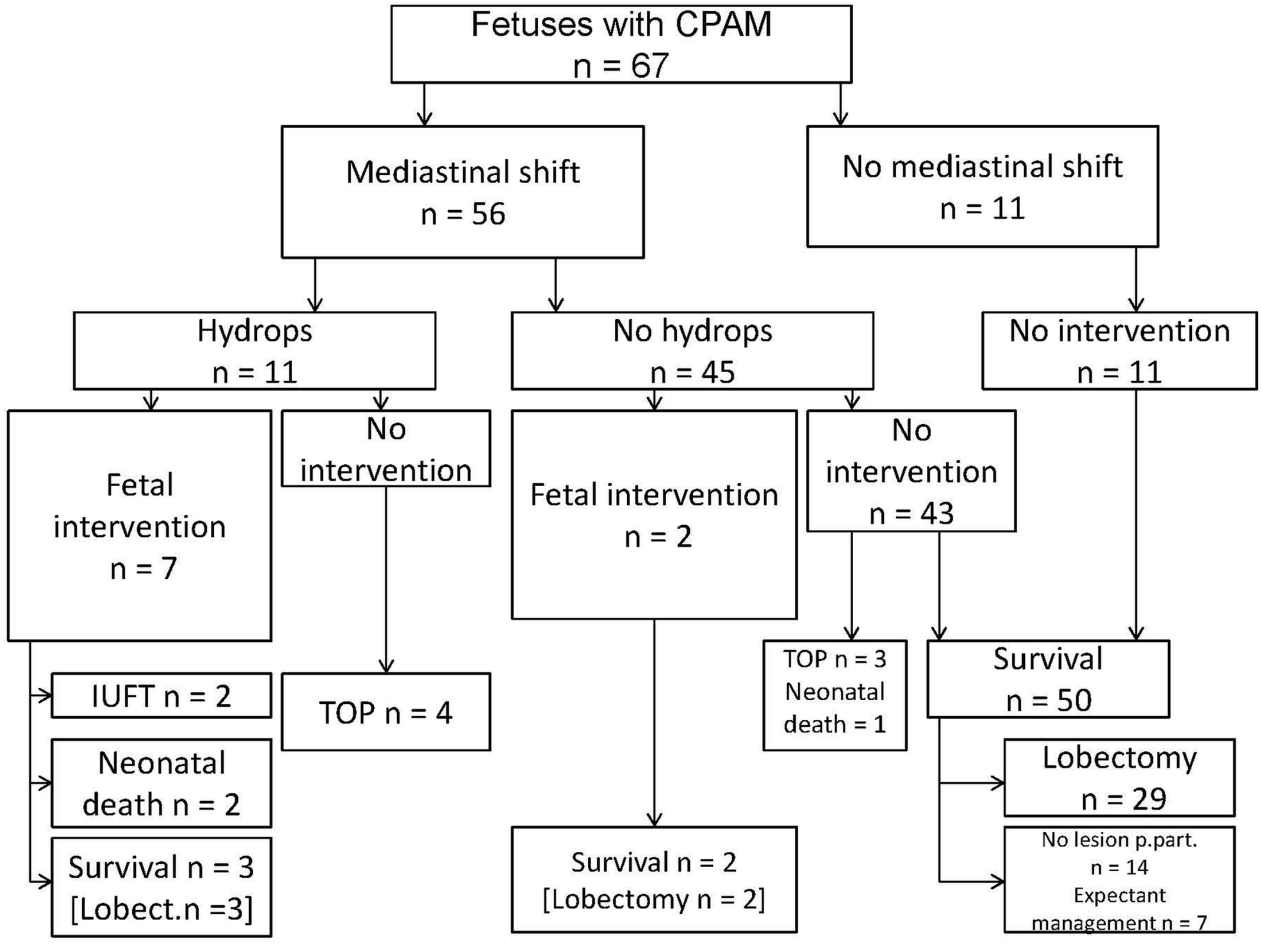
Flow chart showing management and pregnancy outcome of 67 fetuses with prenatally diagnosed CPAM.

Hydropic fetuses with CPAM were observed in 16.4% (11/67 cases). Of these, 81.8% (9/11) were macrocystic and two microcystic. Five further fetuses had unilateral pleural effusions without hydrops (Fig 1).

Karyotyping was performed in 19 (28.4%) cases and revealed trisomy 21 in one case with an additional perimembranous outlet ventricular septal defect. Other additional malformations were noted in 5 (7.5%) cases (two cases with muscular ventricular septal defect, one case with unilateral hydronephrosis, one case with bilateral hydronephrosis and one case with hydrocephalus internus). In 7 cases (10.4%) the parents opted for termination of pregnancy, including the fetus with trisomy 21 and 4 cases with fetal hydrops.

Of the remaining 60 cases, 33 (55%) showed regression in the course of pregnancy (22; 36,6% partial, 11; 18.3% complete). 25 (41.7%) lesions remained unchanged, and two showed progression. Spontaneous regression in cases of uncomplicated CPAM without need for intervention was observed in more than half of continued pregnancies (33/60, 55%), predominantly in microcystic and mixed lesions. In 11 macrocystic lesions none of the cases regressed and progression was noted in two. In contrast, 73.5% (25/34) of microcystic lesions showed partial (41.2%, 14/34) or complete (32.3%, 11/34) regression.

**Fig 2:**
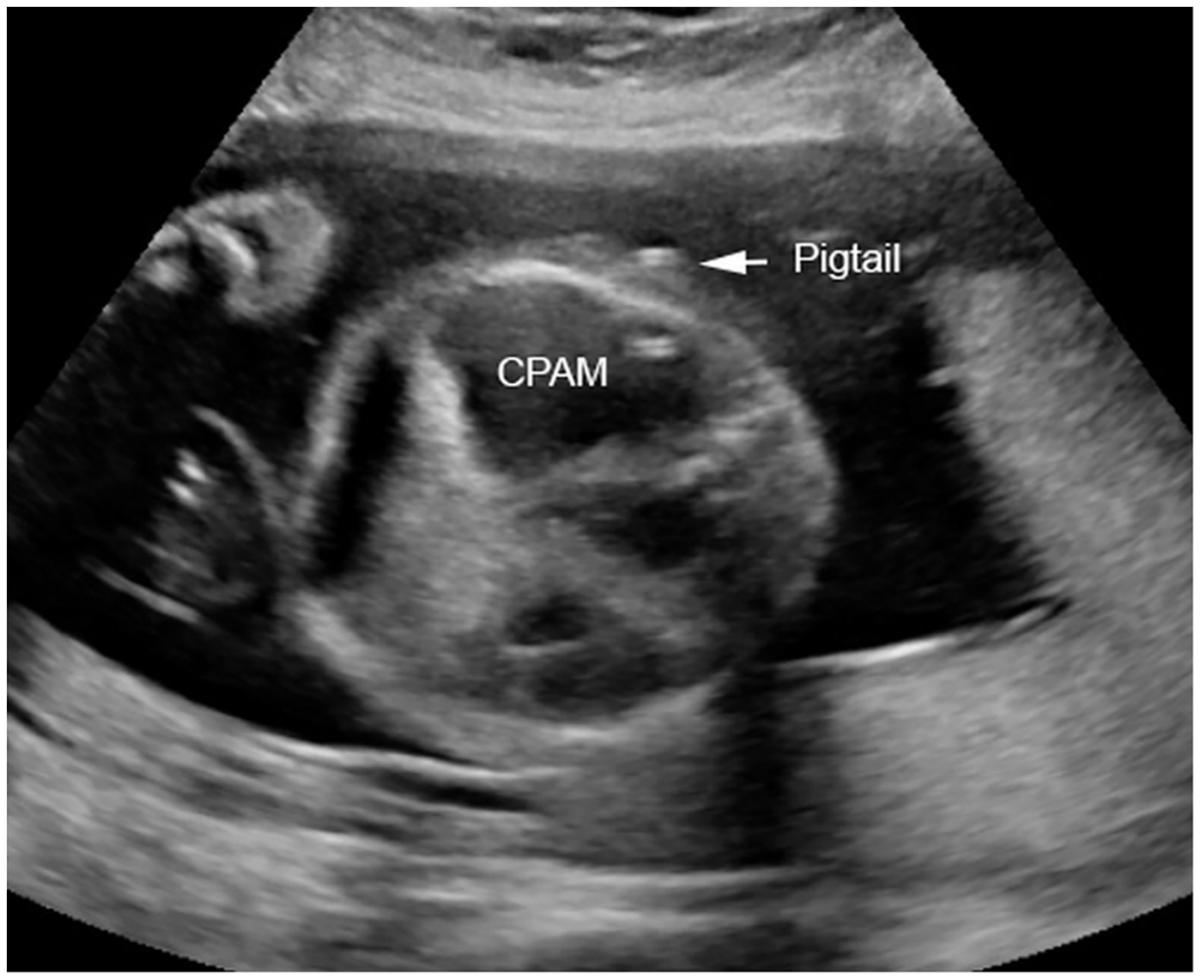
Transverse section of the fetal thorax at 25 weeks of gestation demonstrating a macrocystic congenital pulmonary airway malformation (CPAM) drained by an inserted double pigtail catheter (Harrison Fetal Bladder Stent Set).

9 fetuses (7 of those with hydrops) received an intrauterine intervention: 6 shunt insertions (Fig 2), two instillations of OK-432 and one transplacental treatment with dexamethasone. Besides hydrops inclusion criteria for intervention for the fetuses without hydrops (n = 2) were in one case a large single cyst with moderate mediastinal shift and ascites and in the second case administration of steroids to the mother in presence of severe mediastinal shift due to a large mixed lesion (MTR 0.76, CVR 2.30 and o/e LHR 34.2%), ascites and no possibility of shunting in absence of a large single cyst. Details are given in Table 1.

**Table 1:** Outcome of fetuses with intrauterine treatment of congenital pulmonary airway malformation.

Case	Hydrops	Type of the lesion*	GA (week) at intervention	Type of Intervention	resolution	complication	GA (week) delivery	outcome
**1**	no	I	24	1 cyst puncture	yes	-	39	alive
				instillation of OK-432				
**2**	yes	I	21,22,23,	5 cyst punctures	yes	dislocation of the shunt in 29 weeks	38	alive
			24,27	1 thoraco-amniotic shunt		no reintervention neccessary		
**3**	yes	I	22,23,24	3 cyst punctures	no	IUFT	32	IUFT
				3 instillation of OK-432				
				2 thoraco-amniotic shunts				
**4**	yes	I	25,28,29,30,	2 cyst punctures	yes	dislocation of 4 shunts	36	alive
			32,33,36	7 thoraco-amniotic shunts				
**5**	yes	I	25,28	2 thoraco-amniotic shunts	yes	dislocation of shunt 1	28	neonatal death
						fetal distress after 2nd. intervention, emergency cesarean section		
**6**	yes	I	22	1 thoraco-amniotic shunt	yes	Spontaneous abortion	22	IUFT
**7**	yes	I	28	4 cyst punctures	yes	triplet pregnancy, cesarean section at 31 weeks	31	neonatal death
				1 thoraco-amniotic shunt				
**8**	yes	I	26	1 thoraco-amniotic shunt	yes	-	39	alive
**9**	no	II	22	maternal steroids	yes	-	38	alive

* Classification of Stocker [11],

GA = gestational age, IUFT = intrauterine death

### Pregnancy outcome

The survival rate of fetuses with CPAM was 91.7% (55/60) of those with intention to treat and 98.0% (50/51) for fetuses without hydrops managed conservatively (three cases of TOP excluded). The survival rate for hydropic fetuses with intention to treat was 3/7 (42.9%).

The mean age of delivery for fetuses with CPAM was 37 weeks of gestation (range: 22–40 weeks of gestation). All surviving neonates were born by planned delivery in a tertial center and in presence of neonatologists. 81.8% (45/55) of the neonates showed unremarkable postnatal respiratory function, 10 (18.2%) children needed respiratory assistance. Continuous positive airway pressure was necessary in 5 cases, in 5 cases mechanical ventilation was performed, in no case extracorporal membrane oxygenation-therapy was necessary.

The diagnosis was postnatally confirmed in 64.3% of cases (36/56) by chest X-ray and additional contrast computerized tomography or MRT including one case with early postnatal death. In 2 cases histopathological examination revealed sequestration, in 4 cases congenital lobal emphysema was detected, whereas in all other cases of postnatal surgery (28/34) the diagnosis of CPAM was histologically confirmed. No case of teratoma was detected. In 14 neonates no lesion could be demonstrated in the postpartal period. Postnatal surgery was performed in 61.8% of surviving fetuses (34/55), 7 parents opted for conservative management.

### Sonographic parameters for risk assessment

**Fig 3:**
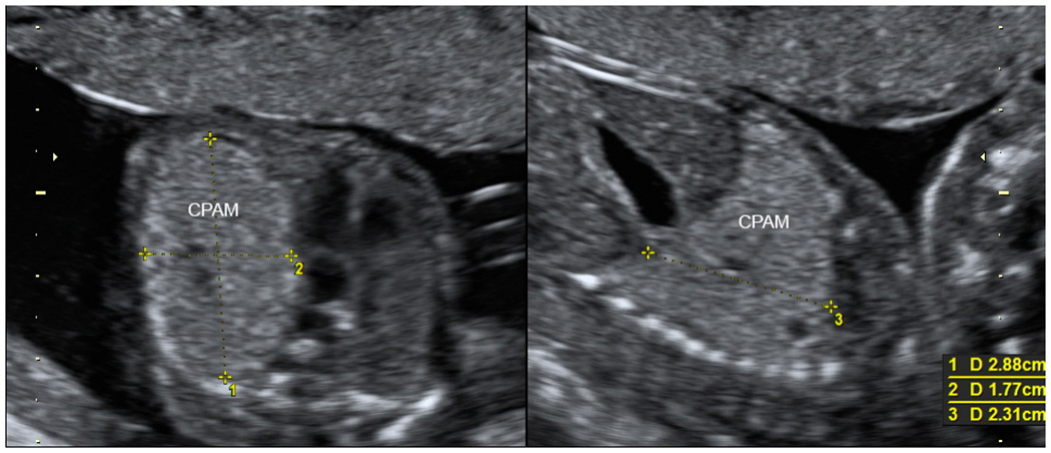
Transverse and longitudinal section of the fetal thorax demonstrating a large microcystic congenital pulmonary airway malformation (CPAM) with mediastinal shift at 19 weeks of gestation. CVR (CPAM volume ratio): 2.88 x 1.77 x 2.31 x 0.52/16.1 (head circumference) = 0.38.

**Fig 4:**
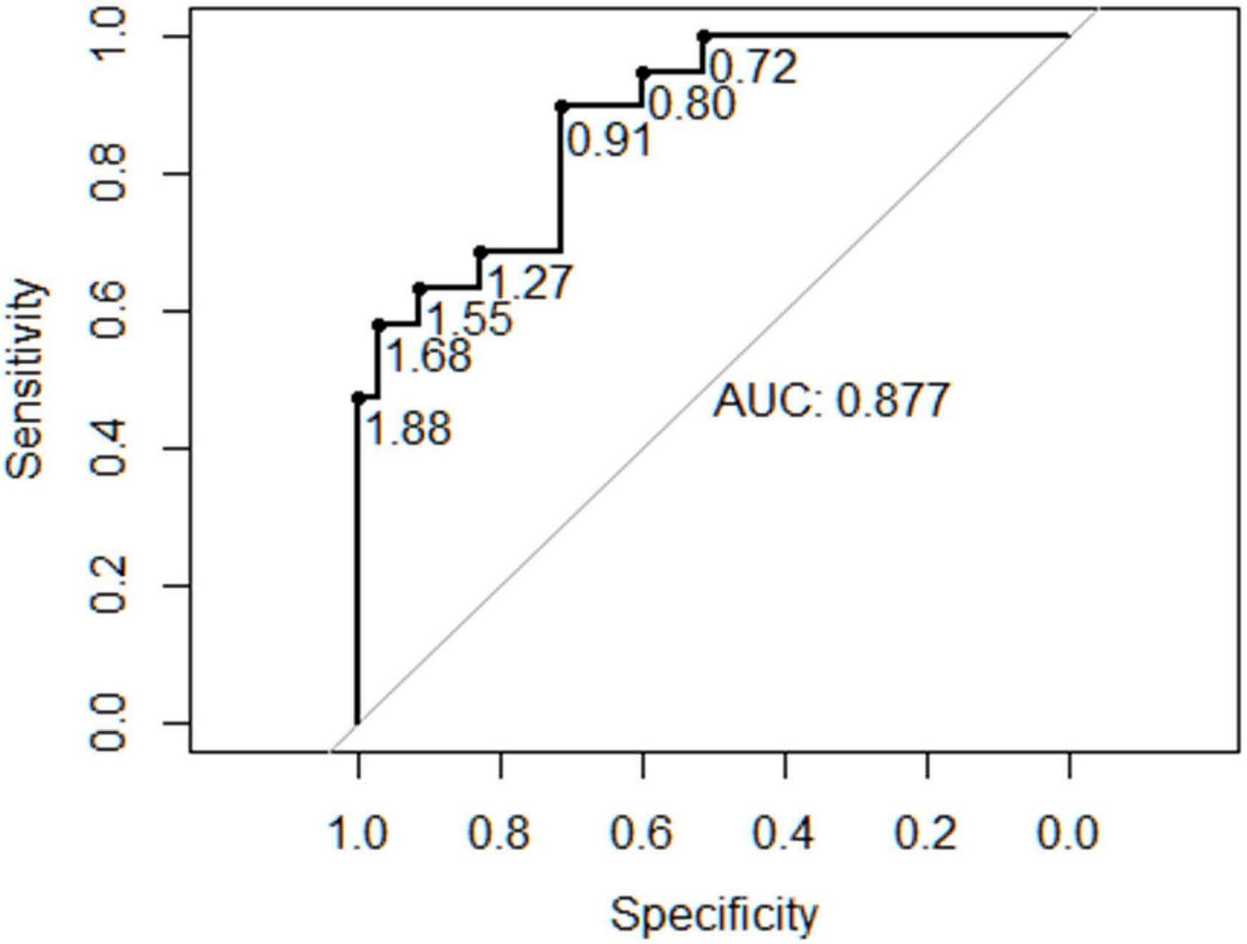
Receiver operator characteristics curve for the congenital pulmonary airway malformation-volume ratio (CVR) and composite adverse outcome or need for intervention.

The CVR measurement (Fig 3), available in 62/67 fetuses, correlated strongly with perinatal death, termination of pregnancy, hydrops, need for prenatal intervention such as pleural drainage and/or thoracocentesis or need for postnatal respiratory assistance (p<0.001). The area under the curve of receiver operator characteristic curves was 0.877 (Fig 4). Fetuses with a CVR of <0.91 were significantly less likely to experience some kind of adverse outcome or need for prenatal intervention with a sensitivity of 0.89 and a specificity of 0.71, a positive predictive value of 0.62 and a negative predictive value of 0.93. A CVR ratio of 1.68 had a sensitivity, specificity, positive predictive value and negative predictive value of 0.58, 0.97, 0.91 and 0.81, respectively.

**Fig 5:**
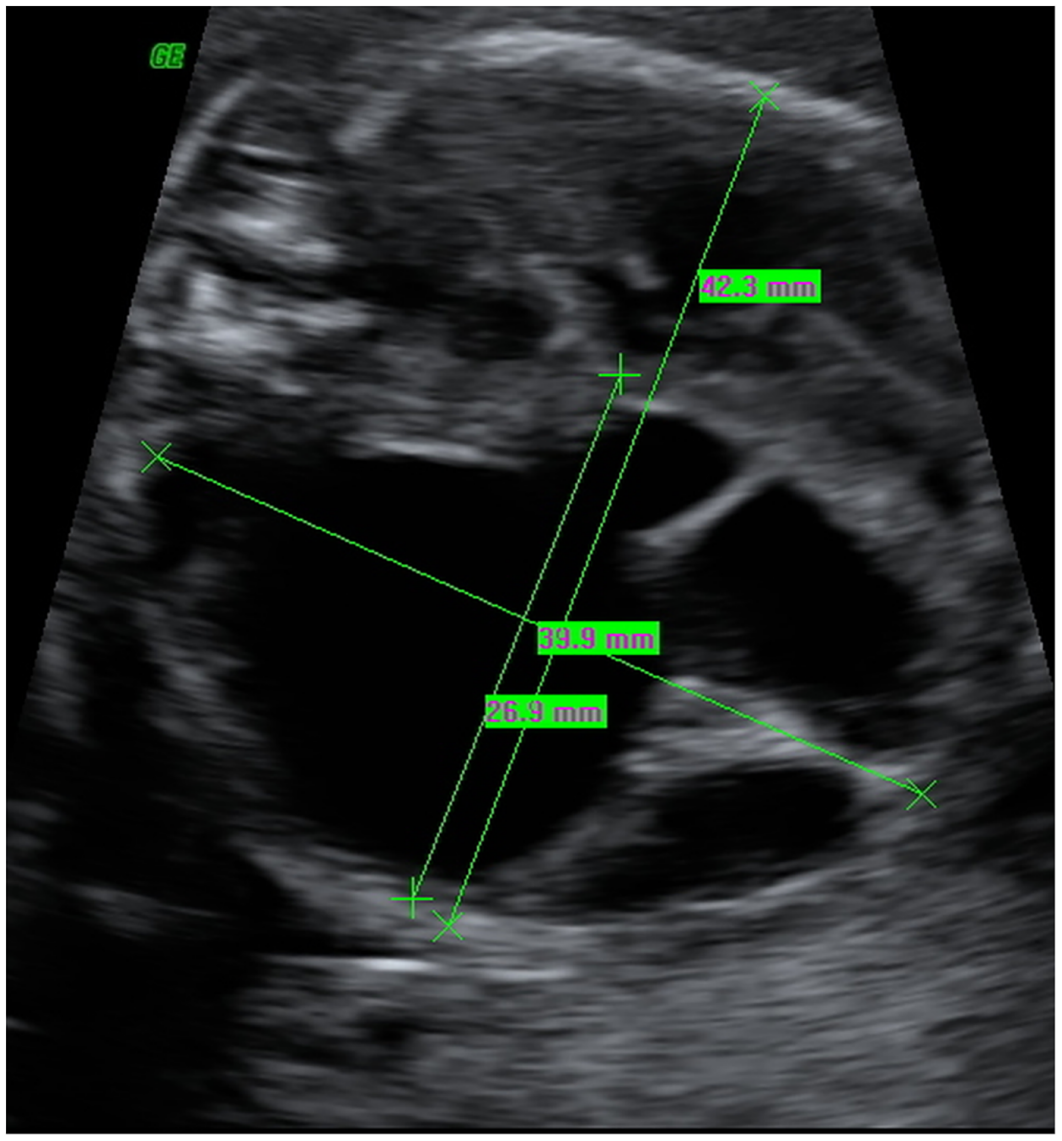
Transverse section of the fetal thorax at 19 weeks of gestation demonstrating a large macrozystic congenital pulmonary airway malformation (CPAM) with severe mediastinal shift. MTR (Mass-to-thorax ratio): 26.9 / 42.3 = 0.63.

**Fig 6:**
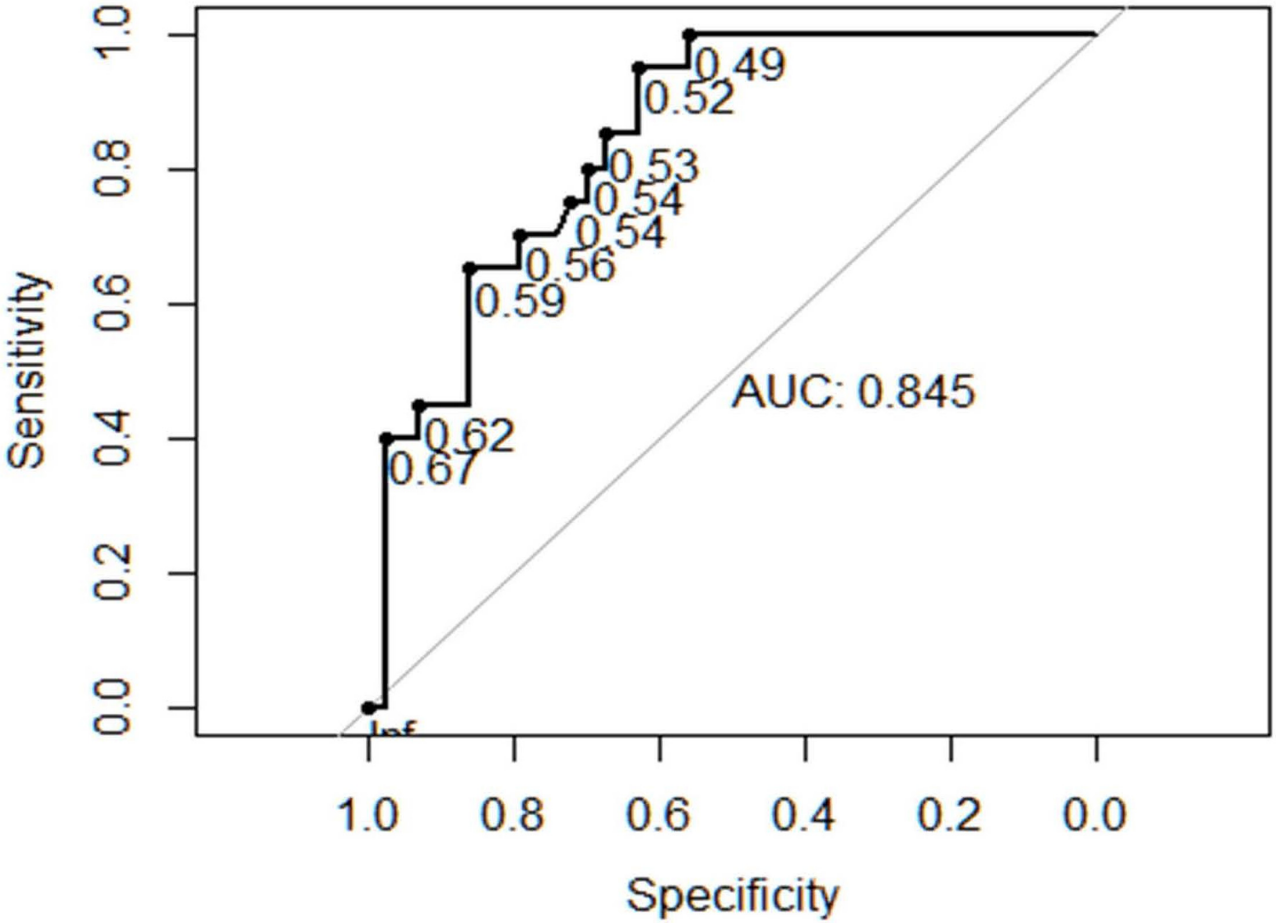
Receiver operator characteristics curve for Mass-to-thorax ratio (MTR) and composite adverse outcome or need for intervention.

As well MTR (mass-to-thorax-ratio) (Fig 5) and adverse outcome were strongly associated (p<0.001). The measurement of the MTR was available in all cases. The area under the curve of receiver operator characteristics curves was 0.845 (Fig 6). A MTR of < 0.51 had a positive predictive value of 0.54 and a negative predictive value of 0.96 of adverse events with a sensitivity of 0.95 and a specificity of 0.63.

**Fig 7:**
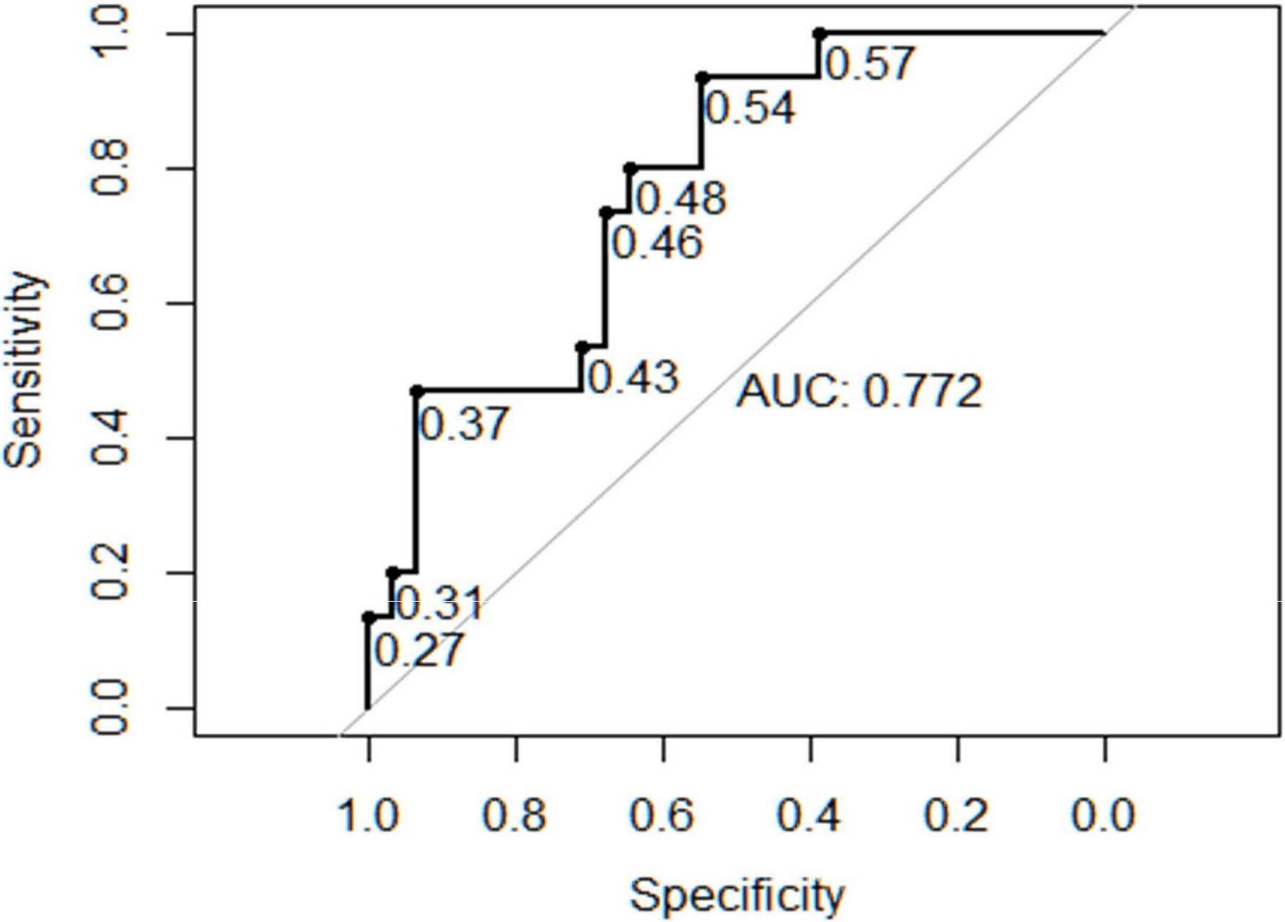
Receiver operator characteristics curve for observed to expected lung-to-head ratio (o/e LHR) and composite adverse outcome or need for intervention.

The measurement of the observed to expected lung area to head circumference (o/e LHR) was less informative than the other two already established prognostic markers, although significant (p = 0.0012). Due to strict criteria of measurement (three images at the exact plane of the four chamber view of the heart) not all of the retrospectively analyzed images could be included (50/67), in one case with hydrocephalus internus and elevated head circumference the ratios could not be calculated. The area under the curve was 0.877 and 0.845 for CVR and MTR in contrast to 0.772 for o/e LHR (Fig 7). An o/e LHR of 45% had a sensitivity of 0.73, a specificity of 0.68 and a positive predictive value of 0.52, a negative predictive value of 0.84 of prediction of adverse outcome. The cutoff value of 25%, which defines fetuses with poor outcome in cases of congenital diaphragmatic hernia, had a positive predictive value of 1.00 with a specificity of 1.00 in the cases with CPAM, but a very low sensitivity of 0.06.

**Fig 8:**
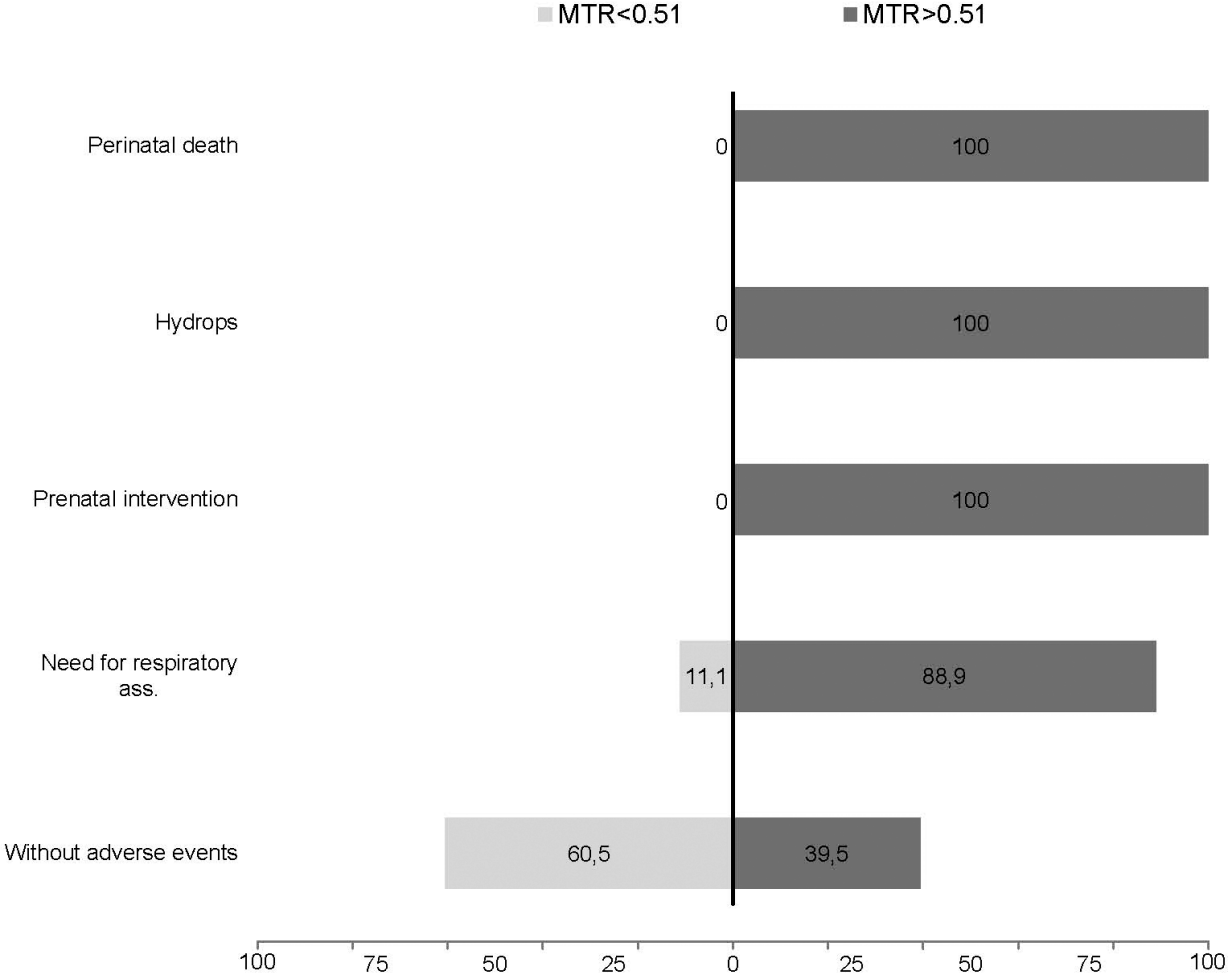
Detectionrate in % of adverse events (perinatal death, hydrops, prenatal intervention or need for respiratory assistance) at a cutoff of 0.51 mass-to-thorax rate (MTR) in comparison to fetuses without adverse events.

**Fig 9:**
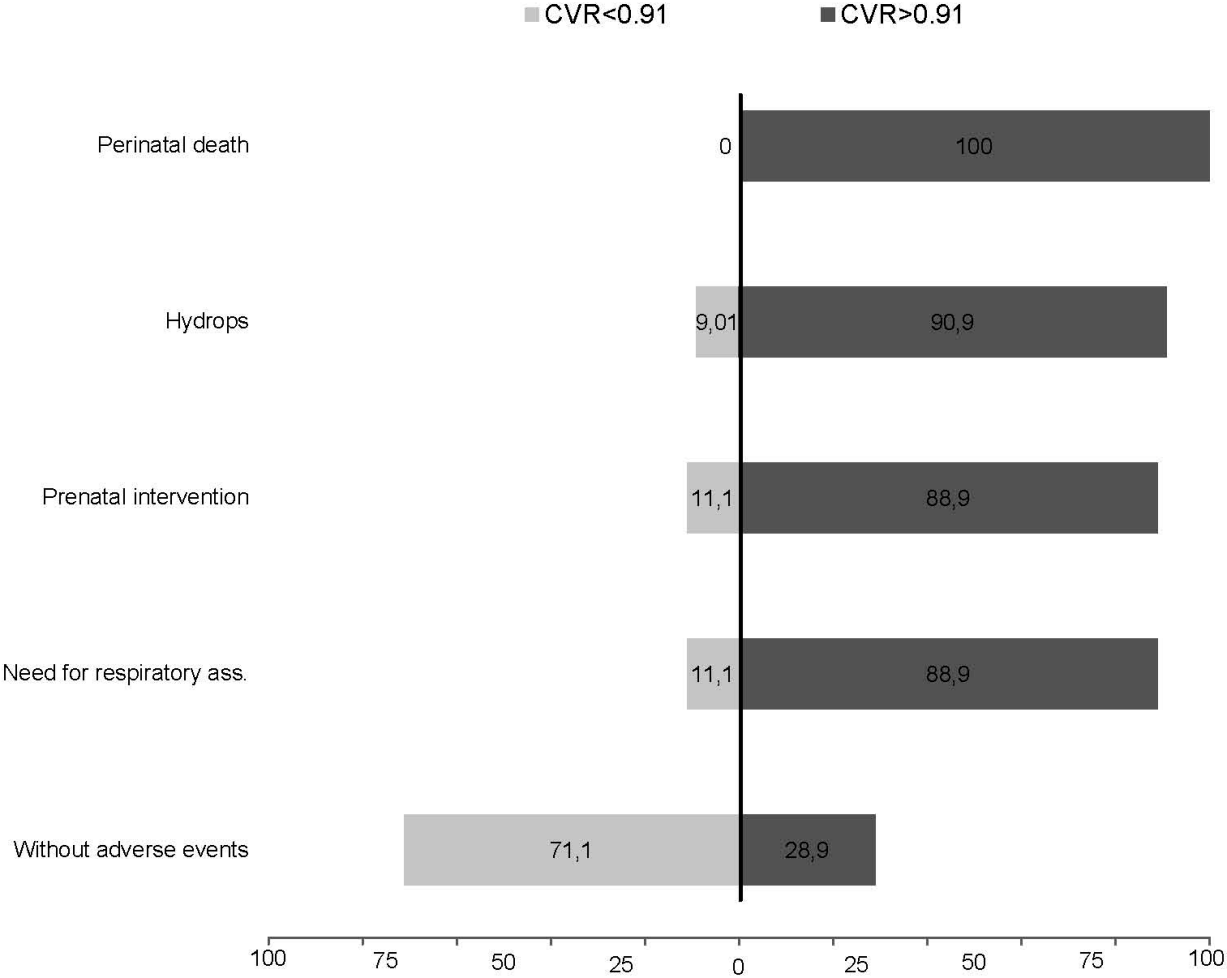
Detectionrate in % of adverse events (perinatal death, hydrops, prenatal intervention or need for respiratory assistance) at a cutoff of 0.91 CPAM volume ratio (CVR) in comparison to fetuses without adverse events.

**Fig 10:**
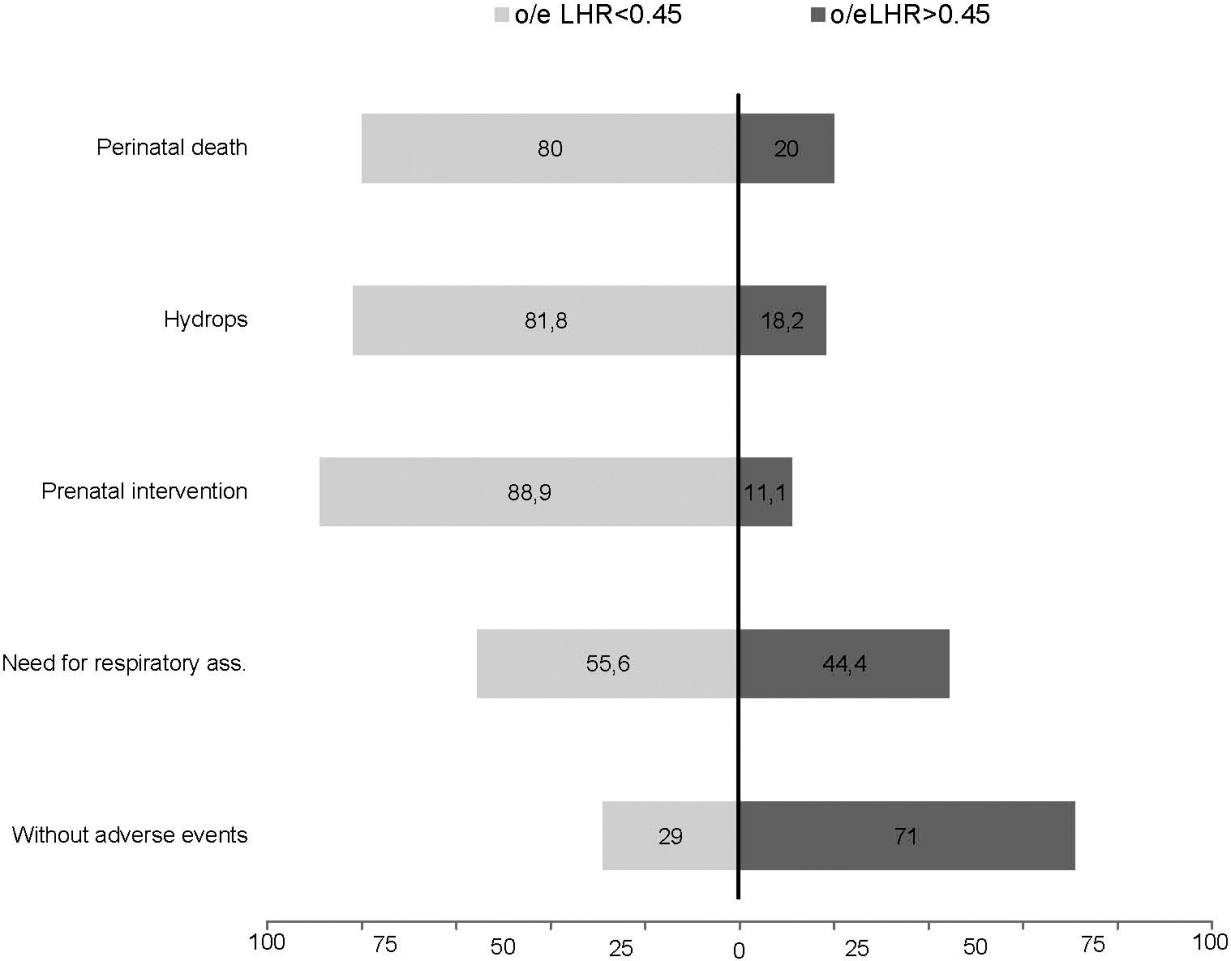
Detectionrate in % of adverse events (perinatal death, hydrops, prenatal intervention or need for respiratory assistance) at a cutoff of 45% observed to expected Lung to head ratio (LHR) in comparison to fetuses without adverse events.

The proportion (%) of cases with each adverse event (perinatal death, hydrops, prenatal intervention, need for respiratory assistance) below and above the selected cutoff (MTR 0.51, CVR 0.91 and o/e LHR 45%) compared to the group of fetuses without adverse events is shown in Figs 8–10.

Logistic regression of a combination of CVR and MTR did not reveal additional information, and a model of the combined score CVR-MTR plus o/e LHR showed no advantage as well.

## Discussion

Congenital pulmonary airway malformation and bronchopulmonary sequestration are the most common lung lesions identified by fetal ultrasound. As ultrasound resolution improves even smaller CPAMs and BPS are now being diagnosed with increasing frequency; the current incident is approximately 1 in 12.000–15.000 live births [14,15]. Moreover Doppler studies have contributed to the distinction between CPAM and BPS by identification of a feeding vessel [16].

CPAM is characterized by abnormal airway patterning during lung branching morphogenesis and is formed by abnormal branching of the immature bronchioles. Growth of the CPAM is unpredictable between 18–26 weeks of gestation. The fastest growth occurs between 20–25 weeks of gestation, a plateau is usually reached at 25 weeks and regression is observed after 29 weeks of gestation [1,3,6,17]. Whereas in the majority of cases the lesion either regresses throughout the pregnancy or remains unchanged and leads to favorable outcome, fetuses with hydrops and severe mediastinal shifting are at risk of intrauterine demise or neonatal death [2,3,12,18,19,20,21,22]. In order to predict fetuses at risk for adverse outcome several sonographic obtained measurements have been established [15,23,24].

Cromblehome *et al*. developed the CPAM volume to head circumference ratio (CVR) to create a gestational age corrected volume ratio for prognostic predication [1,25]. Fetuses with a CVR >1.6 were considered to be at increased risk for developing hydrops. Recently a study of 64 cases of lung lesions found the ratio of CVR >1.6 had a sensitivity of 75% and a positive predictive value of 60%, a negative predictive value of 98% for the detection of hydrops. The authors state that although an initial CVR >1.6 placed a fetus at an increased risk for poorer outcome in general, the cutoff of 1.6 was not sensitive enough to determine fetuses at risk. Therefore they propose a CRV cutoff value of <1.0, predicting a probability of near 100% for being asymptomatic at birth. Yong et al found that even a CVR <0.56 was predictive of good prognosis after birth (negative predictive value 100%) [23]. Our data support these findings suggesting slightly different cutoffs for negative prediction. As reported in the literature a CVR of >1.68 was associated with adverse outcome in our study, but already a CVR of < 0.91 seems to be effective to predict a good outcome with a sensitivity of 89% and a negative predictive value of 93%. In our cohort fetuses with a CVR of <0.56 had a negative predictive value of 1.00 with a sensitivity of 1.00 and a specificity of 0.40 for experiencing some kind of adverse outcome or need for intervention. Summarizing these findings a CVR less than 0.91 calculated at first presentation of the patient predicts a favorable outcome, and sonographic reevaluation can be carried out in larger intervals than in cases with more elevated CVR.

Other prognostic markers for survival of fetuses with CPAM are described by Vu *et al*. who report the outcome of 36 fetuses with large CPAM, including 27 hydropic fetuses [12]. The presence of hydrops was the most important prognostic indicator for poor outcome in fetuses with CPAMs. Additionally Vu *et al*. calculated the mass-to-thorax-ratio (MTR), measured on an axial image of the fetal chest, on which the four-chamber view of the heart was displayed and found that it was highly correlated with the risk of developing hydrops. An MTR cutoff value of 0.56 had an odds ratio of 13.0 for hydrops. Our data support the effectiveness of this marker for prediction of adverse outcome or need for intervention. A MTR of < 0.51 had a positive predictive value of 0.54 and a negative predictive value of 0.96 for adverse events with a sensitivity of 0.95 and a specificity of 0.63. As an easily obtained measurement during a sonographic scan it could be a reassuring tool for predicting outcome and counseling of the parents.

The observed to expected lung area to head ratio (o/e LHR) was first established in the prediction of survival in fetuses with isolated diaphragmatic hernia [13]. The authors found that survival was very poor for fetuses with isolated left sided hernia when the o/e LHR was ≤ 25%. Due to considerable overlap of individual fetuses regarding the value of o/e LHR in affected and normal fetuses, the sensitivity of o/e LHR in the prediction of survival was only 46% with a false positive rate of 10%. Since then the reliability and reproducibility of the o/e LHR was discussed controversial [26,27,28] and compared to fetal MR lung volumetry, which seems to be of slightly better predictive value [29,30]. The o/e LHR has not been used to predict survival in fetuses with intrathoracal lesions as CPAM before, and prognosis or tendency for regression are quite different in CPAM versus congenital diaphragmatic hernia. Whereas the extent of a herniation of intaabdominal organs into the thorax does not regress in the course of pregnancy, CPAM, especially microcystic lesions, have a considerable amount of regression after 29 weeks of gestation. Moreover the survival of fetuses with CDH is remarkably worse than those with CPAM. This might be the reason, that our results regarding LHR are less sensitive as CVR or MTR. Additionally the measurement of o/e LHR was conducted retrospectively in our cohort, which might be less exact than a prospective evaluation.

Regarding other sonographic findings such as the severity of mediastinal shift or presence of polyhydramnios no correlation with the risk of hydrops were found in most studies [12,21,31], whereas macrocystic lesions are known to be associated with hydrops and poor prognosis [1,4,12] probably due to faster expansion of large cysts. Regression of the lesion is reported in 20%-76% in previous series [7,31,32,33], mostly in the microcystic type[2,34]. These findings are congruent to our own observations. The majority of fetuses with CPAM and hydrops had macrocystic lesions with no tendency of regression, whereas regression occurred in 55% in our cohort, predominantly in microcystic lesions.

In cases of non-hydropic fetuses with CPAM the prognosis is excellent with high survival rates and low postnatal morbidity, whereas fetuses with hydrops managed expectantly usually die either before or after delivery [2,3,35]. Therefore numerous attempts of fetal therapy have been made such as thoraco-amniotic shunting or thoracocentesis [5,22,36], prenatal open surgery with hysterotomy and lobectomy [3,6,37] and ultrasound-guided laser ablation [22,38,39], recently described by fetal bronchoscopy[40], or percutaneous radiofrequency [2]. Finally the treatment of fetuses with maternal steriod application is described in small retrospective trials and seems to be effective in microcystic lesions in presence of hydrops, where survival rates with expectant management are described as very low [41,42,43,44]. However, the spontaneous regression rate of microcystic lesions is high and prospective randomized trials are lacking.

Cumulating the recent studies of Cavoretto *et al*. and Lima *et al*. with our own findings results in an excellent survival rate for uncomplicated, conservatively managed CPAM (97.3%, 694/713) [2,45] and also for non-hydropic fetuses treated with thoraco-amniotic shunting (22/25, 88%), whereas hydropic fetuses with thoraco-amniotic shunting and intention to treat survive in about 58.7% (54/92) of cases.

Shunt-related complications such as shunt dislocation or shunt occlusion are described in most series. A recent overview on 286 shunt insertions for treatment of fetal pleural effusion revealed 53 shunt-related complications, of which 18 migrations of the shunt into the thorax or the amniotic cavity were noted[22]. However, in our series shunt dislocation occurred much more frequently (6 /15 procedures), one case presented with four consecutive shunt dislocations. In all cases we used the double pigtail Harrison fetal bladder stent (Cook Medical, Bloomington, IN, USA), which is similar to the Rocket KCH ^™^ Fetal bladder drainage catheter used by other series [46] and provides no anchor system. Recently different types of fetal bladder stents including an anchor system were developed, such as the double basket device [47] or the Somatex intrauterine shunt (Somatex Medical Technologies, Berlin,Germany), which is fixed in the fetal thoracic wall with self-developing parasols. In recent cases of necessity for fetal drainage we use the Somatex sten in order to reduce shunt complications.

Surgical resection is the standard of postnatal care for symptomatic lung lesions; however, the management of asymptomatic CPAM remains controversial [48,49,50,51]. The majority of authors recommend surgical resection to avoid the possible development of complications such as recurrent infection, pneumothorax or rarely development of malignancy [1,4,37,52,53,54,55,56,57,58,59].

In summary the prognosis of antenatally diagnosed CPAM is excellent in the absence of complications such as hydrops fetalis. Fetuses at risk for intrauterine or postnatal death, need for fetal intervention or postnatal respiratory assistance can be detected by CVR, MTR and less sensitive by o/e LHR. Short-term follow up scans, if necessary intrauterine shunting and planned delivery in a tertiary care center are mandatory in these cases.
